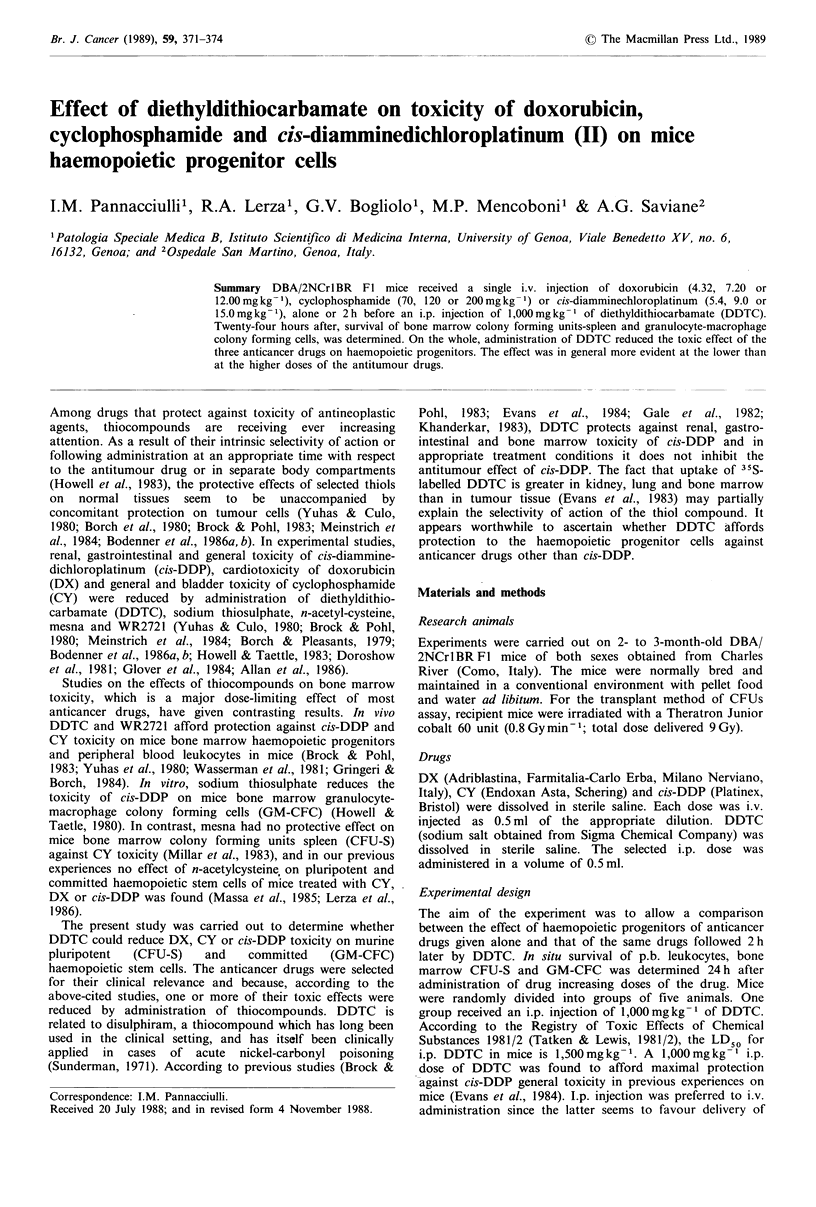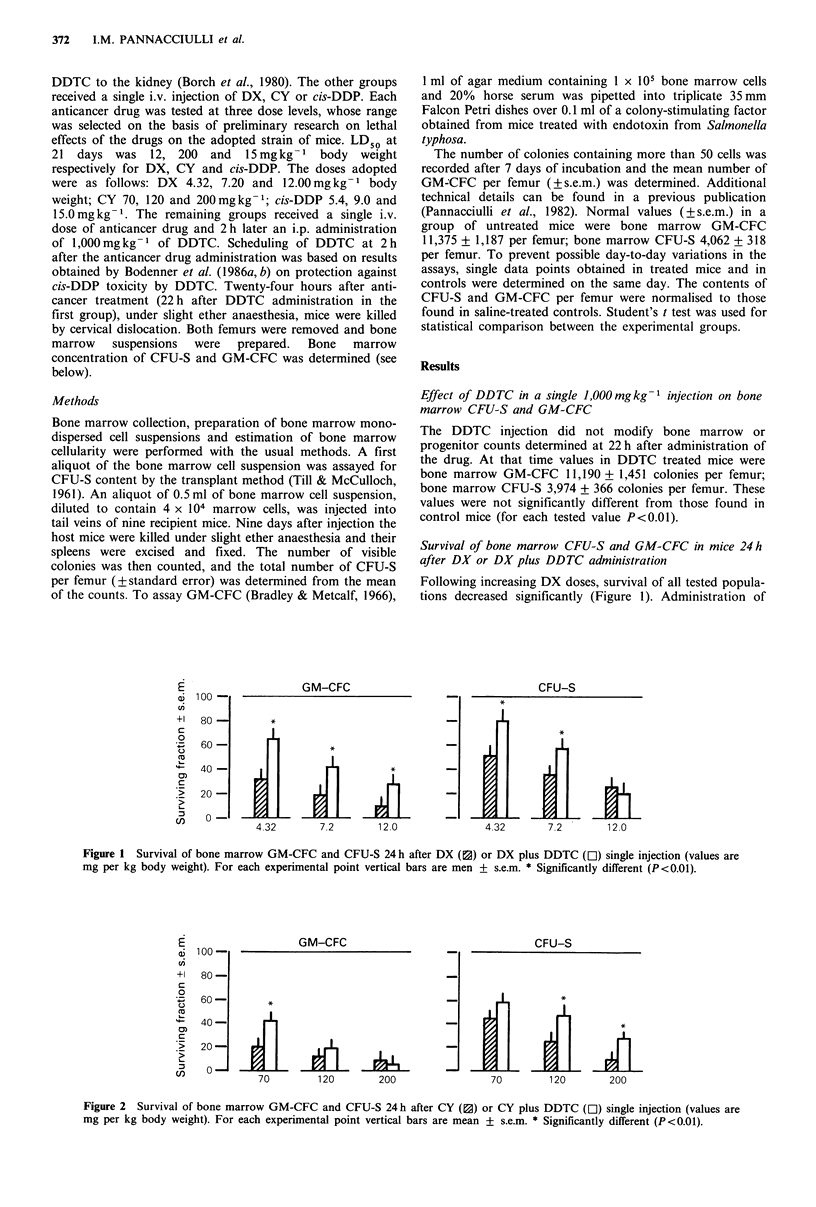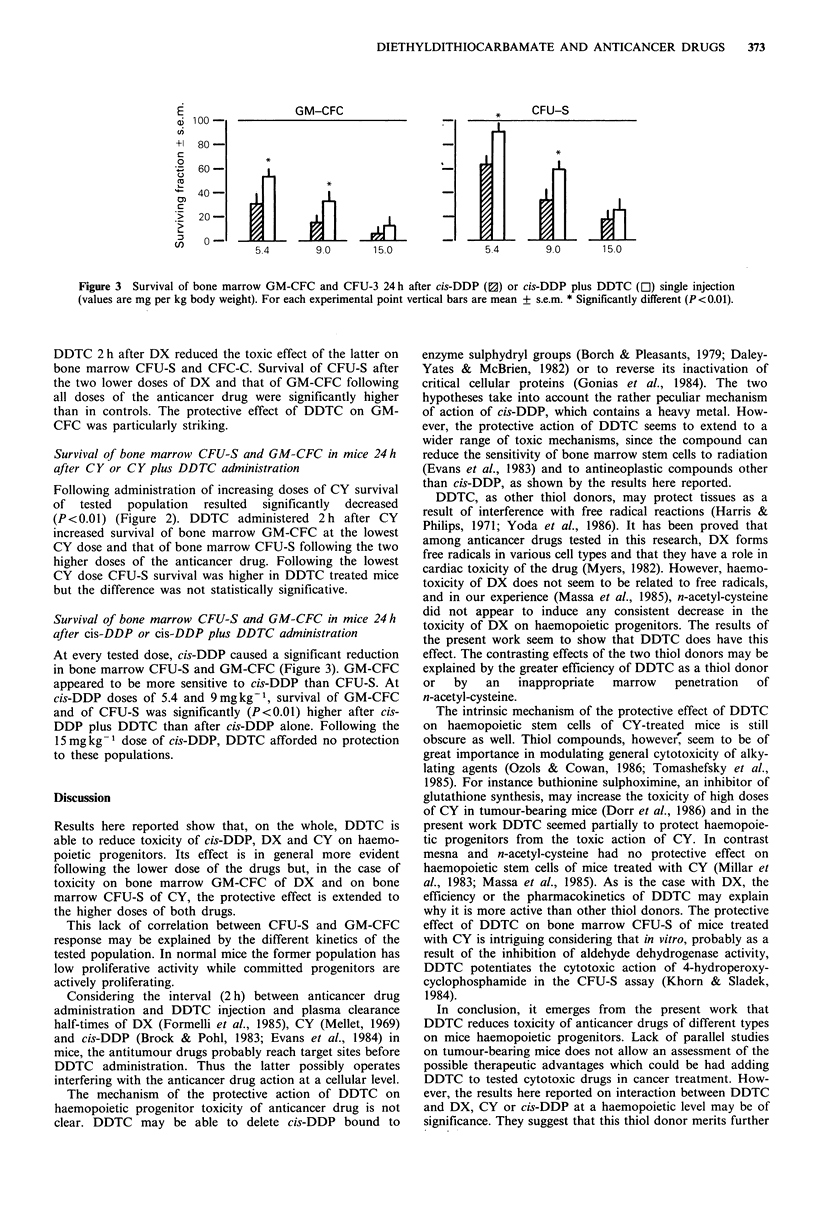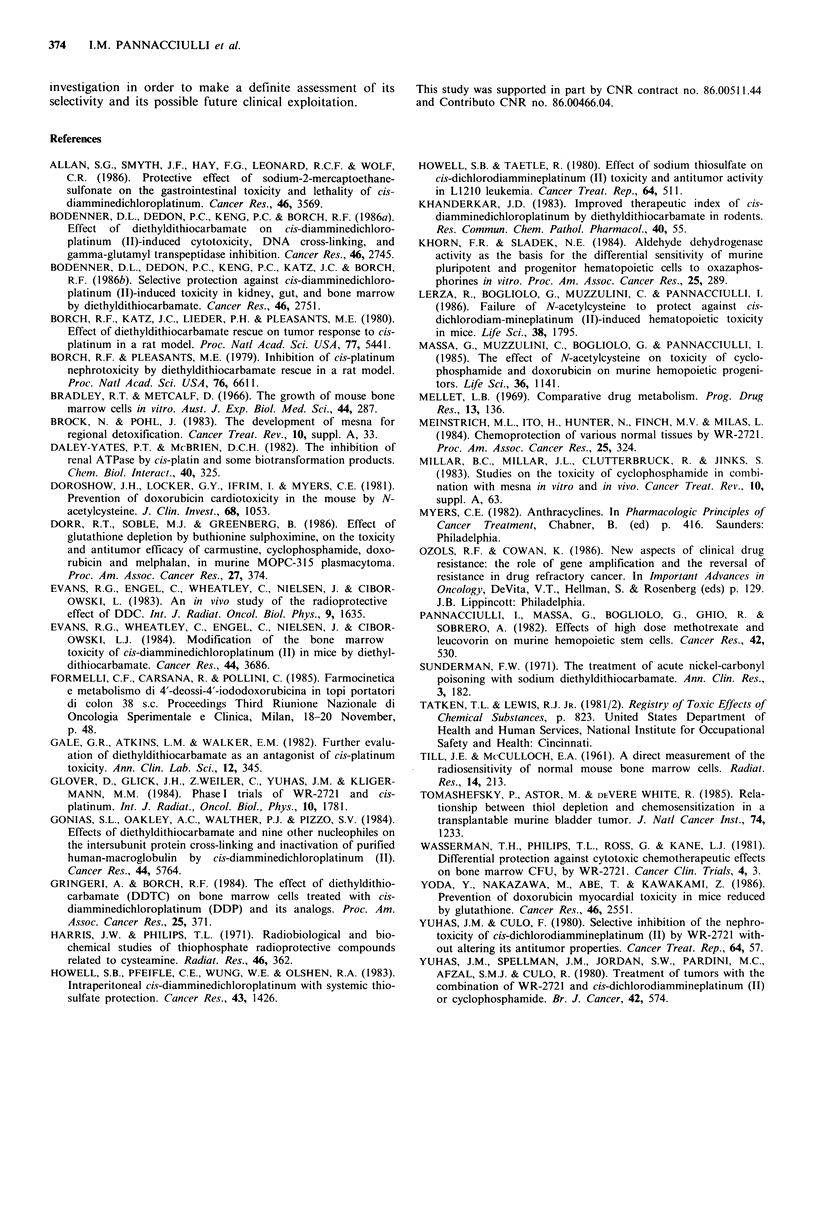# Effect of diethyldithiocarbamate on toxicity of doxorubicin, cyclophosphamide and cis-diamminedichloroplatinum (II) on mice haemopoietic progenitor cells.

**DOI:** 10.1038/bjc.1989.74

**Published:** 1989-03

**Authors:** I. M. Pannacciulli, R. A. Lerza, G. V. Bogliolo, M. P. Mencoboni, A. G. Saviane

**Affiliations:** Patologia Speciale Medica B, Istituto Scientifico di Medicina Interna, University of Genoa, Italy.

## Abstract

DBA/2NCr1BR F1 mice received a single i.v. injection of doxorubicin (4.32, 7.20 or 12.00 mg kg-1), cyclophosphamide (70, 120 or 200 mg kg-1) or cis-diamminechloroplatinum (5.4, 9.0 or 15.0 mg kg-1), alone or 2 h before an i.p. injection of 1,000 mg kg-1 of diethyldithiocarbamate (DDTC). Twenty-four hours after, survival of bone marrow colony forming units-spleen and granulocyte-macrophage colony forming cells, was determined. On the whole, administration of DDTC reduced the toxic effect of the three anticancer drugs on haemopoietic progenitors. The effect was in general more evident at the lower than at the higher doses of the antitumour drugs.


*Br. J. Cancer* (1989), **59**, 371–374 © The Macmillan Press Ltd., 1989

### Effect of diethyldithiocarbamate on toxicity of doxorubicin, cyclophosphamide and *cis*-diamminedichloroplatinum (II) on mice haemopoietic progenitor cells

I.M. Pannacciulli[1], R.A. Lerza[1], G.V. Bogliolo[1], M.P. Mencoboni[1] & A.G. Saviane[2]

[1]*Patologia Speciale Medica B, Istituto Scientifico di Medicina Interna, University of Genoa, Viale Benedetto XV, no. 6, 16132, Genoa; and* [2]*Ospedale San Martino, Genoa, Italy.*

**Summary** DBA/2NCr1BR F1 mice received a single i.v. injection of doxorubicin (4.32, 7.20 or 12.00 mg kg$^{-1}$), cyclophosphamide (70, 120 or 200 mg kg$^{-1}$) or *cis*-diamminechloroplatinum (5.4, 9.0 or 15.0 mg kg$^{-1}$), alone or 2 h before an i.p. injection of 1,000 mg kg$^{-1}$ of diethyldithiocarbamate (DDTC). Twenty-four hours after, survival of bone marrow colony forming units-spleen and granulocyte-macrophage colony forming cells, was determined. On the whole, administration of DDTC reduced the toxic effect of the three anticancer drugs on haemopoietic progenitors. The effect was in general more evident at the lower than at the higher doses of the antitumour drugs.

Among drugs that protect against toxicity of antineoplastic agents, thiocompounds are receiving ever increasing attention. As a result of their intrinsic selectivity of action or following administration at an appropriate time with respect to the antitumour drug or in separate body compartments (Howell *et al.*, 1983), the protective effects of selected thiols on normal tissues seem to be unaccompanied by concomitant protection on tumour cells (Yuhas & Culo, 1980; Borch *et al.*, 1980; Brock & Pohl, 1983; Meinstrich *et al.*, 1984; Bodenner *et al.*, 1986*a*, *b*). In experimental studies, renal, gastrointestinal and general toxicity of *cis*-diamminedichloroplatinum (*cis*-DDP), cardiotoxicity of doxorubicin (DX) and general and bladder toxicity of cyclophosphamide (CY) were reduced by administration of diethyldithiocarbamate (DDTC), sodium thiosulphate, *n*-acetyl-cysteine, mesna and WR2721 (Yuhas & Culo, 1980; Brock & Pohl, 1980; Meinstrich *et al.*, 1984; Borch & Pleasants, 1979; Bodenner *et al.*, 1986*a*, *b*; Howell & Taettle, 1983; Doroshow *et al.*, 1981; Glover *et al.*, 1984; Allan *et al.*, 1986).

Studies on the effects of thiocompounds on bone marrow toxicity, which is a major dose-limiting effect of most anticancer drugs, have given contrasting results. *In vivo* DDTC and WR2721 afford protection against *cis*-DDP and CY toxicity on mice bone marrow haemopoietic progenitors and peripheral blood leukocytes in mice (Brock & Pohl, 1983; Yuhas *et al.*, 1980; Wasserman *et al.*, 1981; Gringeri & Borch, 1984). *In vitro*, sodium thiosulphate reduces the toxicity of *cis*-DDP on mice bone marrow granulocyte-macrophage colony forming cells (GM-CFC) (Howell & Taetle, 1980). In contrast, mesna had no protective effect on mice bone marrow colony forming units spleen (CFU-S) against CY toxicity (Millar *et al.*, 1983), and in our previous experiences no effect of *n*-acetylcysteine on pluripotent and committed haemopoietic stem cells of mice treated with CY, DX or *cis*-DDP was found (Massa *et al.*, 1985; Lerza *et al.*, 1986).

The present study was carried out to determine whether DDTC could reduce DX, CY or *cis*-DDP toxicity on murine pluripotent (CFU-S) and committed (GM-CFC) haemopoietic stem cells. The anticancer drugs were selected for their clinical relevance and because, according to the above-cited studies, one or more of their toxic effects were reduced by administration of thiocompounds. DDTC is related to disulphiram, a thiocompound which has long been used in the clinical setting, and has itself been clinically applied in cases of acute nickel-carbonyl poisoning (Sunderman, 1971). According to previous studies (Brock & Pohl, 1983; Evans *et al.*, 1984; Gale *et al.*, 1982; Khanderkar, 1983), DDTC protects against renal, gastrointestinal and bone marrow toxicity of *cis*-DDP and in appropriate treatment conditions it does not inhibit the antitumour effect of *cis*-DDP. The fact that uptake of $^{35}$S-labelled DDTC is greater in kidney, lung and bone marrow than in tumour tissue (Evans *et al.*, 1983) may partially explain the selectivity of action of the thiol compound. It appears worthwhile to ascertain whether DDTC affords protection to the haemopoietic progenitor cells against anticancer drugs other than *cis*-DDP.

Correspondence: I.M. Pannacciulli.
Received 20 July 1988; and in revised form 4 November 1988.

### Materials and methods

### *Research animals*

Experiments were carried out on 2- to 3-month-old DBA/2NCr1BR F1 mice of both sexes obtained from Charles River (Como, Italy). The mice were normally bred and maintained in a conventional environment with pellet food and water *ad libitum*. For the transplant method of CFUs assay, recipient mice were irradiated with a Theratron Junior cobalt 60 unit (0.8 Gy min$^{-1}$; total dose delivered 9 Gy).

### *Drugs*

DX (Adriblastina, Farmitalia-Carlo Erba, Milano Nerviano, Italy), CY (Endoxan Asta, Schering) and *cis*-DDP (Platinex, Bristol) were dissolved in sterile saline. Each dose was i.v. injected as 0.5 ml of the appropriate dilution. DDTC (sodium salt obtained from Sigma Chemical Company) was dissolved in sterile saline. The selected i.p. dose was administered in a volume of 0.5 ml.

### *Experimental design*

The aim of the experiment was to allow a comparison between the effect of haemopoietic progenitors of anticancer drugs given alone and that of the same drugs followed 2 h later by DDTC. *In situ* survival of p.b. leukocytes, bone marrow CFU-S and GM-CFC was determined 24 h after administration of drug increasing doses of the drug. Mice were randomly divided into groups of five animals. One group received an i.p. injection of 1,000 mg kg$^{-1}$ of DDTC. According to the Registry of Toxic Effects of Chemical Substances 1981/2 (Tatken & Lewis, 1981/2), the $LD_{50}$ for i.p. DDTC in mice is 1,500 mg kg$^{-1}$. A 1,000 mg kg$^{-1}$ i.p. dose of DDTC was found to afford maximal protection against *cis*-DDP general toxicity in previous experiences on mice (Evans *et al.*, 1984). I.p. injection was preferred to i.v. administration since the latter seems to favour delivery of

DDTC to the kidney (Borch et al., 1980). The other groups received a single i.v. injection of DX, CY or *cis*-DDP. Each anticancer drug was tested at three dose levels, whose range was selected on the basis of preliminary research on lethal effects of the drugs on the adopted strain of mice. $LD_{50}$ at 21 days was 12, 200 and 15 mg kg$^{-1}$ body weight respectively for DX, CY and *cis*-DDP. The doses adopted were as follows: DX 4.32, 7.20 and 12.00 mg kg$^{-1}$ body weight; CY 70, 120 and 200 mg kg$^{-1}$; *cis*-DDP 5.4, 9.0 and 15.0 mg kg$^{-1}$. The remaining groups received a single i.v. dose of anticancer drug and 2 h later an i.p. administration of 1,000 mg kg$^{-1}$ of DDTC. Scheduling of DDTC at 2 h after the anticancer drug administration was based on results obtained by Bodenner et al. (1986*a*, *b*) on protection against *cis*-DDP toxicity by DDTC. Twenty-four hours after anticancer treatment (22 h after DDTC administration in the first group), under slight ether anaesthesia, mice were killed by cervical dislocation. Both femurs were removed and bone marrow suspensions were prepared. Bone marrow concentration of CFU-S and GM-CFC was determined (see below).

### *Methods*

Bone marrow collection, preparation of bone marrow monodispersed cell suspensions and estimation of bone marrow cellularity were performed with the usual methods. A first aliquot of the bone marrow cell suspension was assayed for CFU-S content by the transplant method (Till & McCulloch, 1961). An aliquot of 0.5 ml of bone marrow cell suspension, diluted to contain $4 \times 10^4$ marrow cells, was injected into tail veins of nine recipient mice. Nine days after injection the host mice were killed under slight ether anaesthesia and their spleens were excised and fixed. The number of visible colonies was then counted, and the total number of CFU-S per femur (±standard error) was determined from the mean of the counts. To assay GM-CFC (Bradley & Metcalf, 1966), 1 ml of agar medium containing $1 \times 10^5$ bone marrow cells and 20% horse serum was pipetted into triplicate 35 mm Falcon Petri dishes over 0.1 ml of a colony-stimulating factor obtained from mice treated with endotoxin from *Salmonella typhosa*.

The number of colonies containing more than 50 cells was recorded after 7 days of incubation and the mean number of GM-CFC per femur (±s.e.m.) was determined. Additional technical details can be found in a previous publication (Pannacciulli et al., 1982). Normal values (±s.e.m.) in a group of untreated mice were bone marrow GM-CFC 11,375 ± 1,187 per femur; bone marrow CFU-S 4,062 ± 318 per femur. To prevent possible day-to-day variations in the assays, single data points obtained in treated mice and in controls were determined on the same day. The contents of CFU-S and GM-CFC per femur were normalised to those found in saline-treated controls. Student's *t* test was used for statistical comparison between the experimental groups.

### Results

### *Effect of DDTC in a single 1,000 mg kg$^{-1}$ injection on bone marrow CFU-S and GM-CFC*

The DDTC injection did not modify bone marrow or progenitor counts determined at 22 h after administration of the drug. At that time values in DDTC treated mice were bone marrow GM-CFC 11,190 ± 1,451 colonies per femur; bone marrow CFU-S 3,974 ± 366 colonies per femur. These values were not significantly different from those found in control mice (for each tested value $P<0.01$).

### *Survival of bone marrow CFU-S and GM-CFC in mice 24 h after DX or DX plus DDTC administration*

Following increasing DX doses, survival of all tested populations decreased significantly (Figure 1). Administration of

**Figure 1** Survival of bone marrow GM-CFC and CFU-S 24 h after DX (▨) or DX plus DDTC (□) single injection (values are mg per kg body weight). For each experimental point vertical bars are men ± s.e.m. * Significantly different ($P<0.01$).

**Figure 2** Survival of bone marrow GM-CFC and CFU-S 24 h after CY (▨) or CY plus DDTC (□) single injection (values are mg per kg body weight). For each experimental point vertical bars are mean ± s.e.m. * Significantly different ($P<0.01$).

**Figure 3** Survival of bone marrow GM-CFC and CFU-3 24 h after *cis*-DDP (▨) or *cis*-DDP plus DDTC (□) single injection (values are mg per kg body weight). For each experimental point vertical bars are mean ± s.e.m. * Significantly different ($P<0.01$).

DDTC 2 h after DX reduced the toxic effect of the latter on bone marrow CFU-S and CFC-C. Survival of CFU-S after the two lower doses of DX and that of GM-CFC following all doses of the anticancer drug were significantly higher than in controls. The protective effect of DDTC on GM-CFC was particularly striking.

*Survival of bone marrow CFU-S and GM-CFC in mice 24 h after CY or CY plus DDTC administration*

Following administration of increasing doses of CY survival of tested population resulted significantly decreased ($P<0.01$) (Figure 2). DDTC administered 2 h after CY increased survival of bone marrow GM-CFC at the lowest CY dose and that of bone marrow CFU-S following the two higher doses of the anticancer drug. Following the lowest CY dose CFU-S survival was higher in DDTC treated mice but the difference was not statistically significative.

*Survival of bone marrow CFU-S and GM-CFC in mice 24 h after* cis-*DDP or* cis-*DDP plus DDTC administration*

At every tested dose, *cis*-DDP caused a significant reduction in bone marrow CFU-S and GM-CFC (Figure 3). GM-CFC appeared to be more sensitive to *cis*-DDP than CFU-S. At *cis*-DDP doses of 5.4 and 9 $mg\,kg^{-1}$, survival of GM-CFC and of CFU-S was significantly ($P<0.01$) higher after *cis*-DDP plus DDTC than after *cis*-DDP alone. Following the 15 $mg\,kg^{-1}$ dose of *cis*-DDP, DDTC afforded no protection to these populations.

### Discussion

Results here reported show that, on the whole, DDTC is able to reduce toxicity of *cis*-DDP, DX and CY on haemopoietic progenitors. Its effect is in general more evident following the lower dose of the drugs but, in the case of toxicity on bone marrow GM-CFC of DX and on bone marrow CFU-S of CY, the protective effect is extended to the higher doses of both drugs.

This lack of correlation between CFU-S and GM-CFC response may be explained by the different kinetics of the tested population. In normal mice the former population has low proliferative activity while committed progenitors are actively proliferating.

Considering the interval (2 h) between anticancer drug administration and DDTC injection and plasma clearance half-times of DX (Formelli *et al.*, 1985), CY (Mellet, 1969) and *cis*-DDP (Brock & Pohl, 1983; Evans *et al.*, 1984) in mice, the antitumour drugs probably reach target sites before DDTC administration. Thus the latter possibly operates interfering with the anticancer drug action at a cellular level.

The mechanism of the protective action of DDTC on haemopoietic progenitor toxicity of anticancer drug is not clear. DDTC may be able to delete *cis*-DDP bound to enzyme sulphydryl groups (Borch & Pleasants, 1979; Daley-Yates & McBrien, 1982) or to reverse its inactivation of critical cellular proteins (Gonias *et al.*, 1984). The two hypotheses take into account the rather peculiar mechanism of action of *cis*-DDP, which contains a heavy metal. However, the protective action of DDTC seems to extend to a wider range of toxic mechanisms, since the compound can reduce the sensitivity of bone marrow stem cells to radiation (Evans *et al.*, 1983) and to antineoplastic compounds other than *cis*-DDP, as shown by the results here reported.

DDTC, as other thiol donors, may protect tissues as a result of interference with free radical reactions (Harris & Philips, 1971; Yoda *et al.*, 1986). It has been proved that among anticancer drugs tested in this research, DX forms free radicals in various cell types and that they have a role in cardiac toxicity of the drug (Myers, 1982). However, haemotoxicity of DX does not seem to be related to free radicals, and in our experience (Massa *et al.*, 1985), *n*-acetyl-cysteine did not appear to induce any consistent decrease in the toxicity of DX on haemopoietic progenitors. The results of the present work seem to show that DDTC does have this effect. The contrasting effects of the two thiol donors may be explained by the greater efficiency of DDTC as a thiol donor or by an inappropriate marrow penetration of *n*-acetyl-cysteine.

The intrinsic mechanism of the protective effect of DDTC on haemopoietic stem cells of CY-treated mice is still obscure as well. Thiol compounds, however, seem to be of great importance in modulating general cytotoxicity of alkylating agents (Ozols & Cowan, 1986; Tomashefsky *et al.*, 1985). For instance buthionine sulphoximine, an inhibitor of glutathione synthesis, may increase the toxicity of high doses of CY in tumour-bearing mice (Dorr *et al.*, 1986) and in the present work DDTC seemed partially to protect haemopoietic progenitors from the toxic action of CY. In contrast mesna and *n*-acetyl-cysteine had no protective effect on haemopoietic stem cells of mice treated with CY (Millar *et al.*, 1983; Massa *et al.*, 1985). As is the case with DX, the efficiency or the pharmacokinetics of DDTC may explain why it is more active than other thiol donors. The protective effect of DDTC on bone marrow CFU-S of mice treated with CY is intriguing considering that *in vitro*, probably as a result of the inhibition of aldehyde dehydrogenase activity, DDTC potentiates the cytotoxic action of 4-hydroperoxy-cyclophosphamide in the CFU-S assay (Khorn & Sladek, 1984).

In conclusion, it emerges from the present work that DDTC reduces toxicity of anticancer drugs of different types on mice haemopoietic progenitors. Lack of parallel studies on tumour-bearing mice does not allow an assessment of the possible therapeutic advantages which could be had adding DDTC to tested cytotoxic drugs in cancer treatment. However, the results here reported on interaction between DDTC and DX, CY or *cis*-DDP at a haemopoietic level may be of significance. They suggest that this thiol donor merits further investigation in order to make a definite assessment of its selectivity and its possible future clinical exploitation.

This study was supported in part by CNR contract no. 86.00511.44 and Contributo CNR no. 86.00466.04.

### References

ALLAN, S.G., SMYTH, J.F., HAY, F.G., LEONARD, R.C.F. & WOLF, C.R. (1986). Protective effect of sodium-2-mercaptoethane-sulfonate on the gastrointestinal toxicity and lethality of *cis*-diamminedichloroplatinum. *Cancer Res.*, **46**, 3569.

BODENNER, D.L., DEDON, P.C., KENG, P.C. & BORCH, R.F. (1986*a*). Effect of diethyldithiocarbamate on *cis*-diamminedichloroplatinum (II)-induced cytotoxicity, DNA cross-linking, and gamma-glutamyl transpeptidase inhibition. *Cancer Res.*, **46**, 2745.

BODENNER, D.L., DEDON, P.C., KENG, P.C., KATZ, J.C. & BORCH, R.F. (1986*b*). Selective protection against *cis*-diamminedichloroplatinum (II)-induced toxicity in kidney, gut, and bone marrow by diethyldithiocarbamate. *Cancer Res.*, **46**, 2751.

BORCH, R.F., KATZ, J.C., LIEDER, P.H. & PLEASANTS, M.E. (1980). Effect of diethyldithiocarbamate rescue on tumor response to *cis*-platinum in a rat model. *Proc. Natl Acad. Sci. USA*, **77**, 5441.

BORCH, R.F. & PLEASANTS, M.E. (1979). Inhibition of *cis*-platinum nephrotoxicity by diethyldithiocarbamate rescue in a rat model. *Proc. Natl Acad. Sci. USA*, **76**, 6611.

BRADLEY, R.T. & METCALF, D. (1966). The growth of mouse bone marrow cells *in vitro*. *Aust. J. Exp. Biol. Med. Sci.*, **44**, 287.

BROCK, N. & POHL, J. (1983). The development of mesna for regional detoxification. *Cancer Treat. Rev.*, **10**, suppl. A, 33.

DALEY-YATES, P.T. & McBRIEN, D.C.H. (1982). The inhibition of renal ATPase by *cis*-platin and some biotransformation products. *Chem. Biol. Interact.*, **40**, 325.

DOROSHOW, J.H., LOCKER, G.Y., IFRIM, I. & MYERS, C.E. (1981). Prevention of doxorubicin cardiotoxicity in the mouse by *N*-acetylcysteine. *J. Clin. Invest.*, **68**, 1053.

DORR, R.T., SOBLE, M.J. & GREENBERG, B. (1986). Effect of glutathione depletion by buthionine sulphoximine, on the toxicity and antitumor efficacy of carmustine, cyclophosphamide, doxorubicin and melphalan, in murine MOPC-315 plasmacytoma. *Proc. Am. Assoc. Cancer Res.*, **27**, 374.

EVANS, R.G., ENGEL, C., WHEATLEY, C., NIELSEN, J. & CIBOROWSKI, L. (1983). An *in vivo* study of the radioprotective effect of DDC. *Int. J. Radiat. Oncol. Biol. Phys.*, **9**, 1635.

EVANS, R.G., WHEATLEY, C., ENGEL, C., NIELSEN, J. & CIBOROWSKI, L.J. (1984). Modification of the bone marrow toxicity of *cis*-diamminedichloroplatinum (II) in mice by diethyldithiocarbamate. *Cancer Res.*, **44**, 3686.

FORMELLI, C.F., CARSANA, R. & POLLINI, C. (1985). Farmocinetica e metabolismo di 4′-deossi-4′-iododoxorubicina in topi portatori di colon 38 s.c. Proceedings Third Riunione Nazionale di Oncologia Sperimentale e Clinica, Milan, 18–20 November, p. 48.

GALE, G.R., ATKINS, L.M. & WALKER, E.M. (1982). Further evaluation of diethyldithiocarbamate as an antagonist of *cis*-platinum toxicity. *Ann. Clin. Lab. Sci.*, **12**, 345.

GLOVER, D., GLICK, J.H., Z.WEILER, C., YUHAS, J.M. & KLIGERMANN, M.M. (1984). Phase I trials of WR-2721 and *cis*-platinum. *Int. J. Radiat., Oncol. Biol., Phys.*, **10**, 1781.

GONIAS, S.L., OAKLEY, A.C., WALTHER, P.J. & PIZZO, S.V. (1984). Effects of diethyldithiocarbamate and nine other nucleophiles on the intersubunit protein cross-linking and inactivation of purified human-macroglobulin by *cis*-diamminedichloroplatinum (II). *Cancer Res.*, **44**, 5764.

GRINGERI, A. & BORCH, R.F. (1984). The effect of diethyldithiocarbamate (DDTC) on bone marrow cells treated with *cis*-diamminedichloroplatinum (DDP) and its analogs. *Proc. Am. Assoc. Cancer Res.*, **25**, 371.

HARRIS, J.W. & PHILIPS, T.L. (1971). Radiobiological and biochemical studies of thiophosphate radioprotective compounds related to cysteamine. *Radiat. Res.*, **46**, 362.

HOWELL, S.B., PFEIFLE, C.E., WUNG, W.E. & OLSHEN, R.A. (1983). Intraperitoneal *cis*-diamminedichloroplatinum with systemic thiosulfate protection. *Cancer Res.*, **43**, 1426.

HOWELL, S.B. & TAETLE, R. (1980). Effect of sodium thiosulfate on *cis*-dichlorodiammineplatinum (II) toxicity and antitumor activity in L1210 leukemia. *Cancer Treat. Rep.*, **64**, 511.

KHANDERKAR, J.D. (1983). Improved therapeutic index of *cis*-diamminedichloroplatinum by diethyldithiocarbamate in rodents. *Res. Commun. Chem. Pathol. Pharmacol.*, **40**, 55.

KHORN, F.R. & SLADEK, N.E. (1984). Aldehyde dehydrogenase activity as the basis for the differential sensitivity of murine pluripotent and progenitor hematopoietic cells to oxazaphosphorines *in vitro*. *Proc. Am. Assoc. Cancer Res.*, **25**, 289.

LERZA, R., BOGLIOLO, G., MUZZULINI, C. & PANNACCIULLI, I. (1986). Failure of *N*-acetylcysteine to protect against *cis*-dichlorodiam-mineplatinum (II)-induced hematopoietic toxicity in mice. *Life Sci.*, **38**, 1795.

MASSA, G., MUZZULINI, C., BOGLIOLO, G. & PANNACCIULLI, I. (1985). The effect of *N*-acetylcysteine on toxicity of cyclophosphamide and doxorubicin on murine hemopoietic progenitors. *Life Sci.*, **36**, 1141.

MELLET, L.B. (1969). Comparative drug metabolism. *Prog. Drug Res.*, **13**, 136.

MEINSTRICH, M.L., ITO, H., HUNTER, N., FINCH, M.V. & MILAS, L. (1984). Chemoprotection of various normal tissues by WR-2721. *Proc. Am. Assoc. Cancer Res.*, **25**, 324.

MILLAR, B.C., MILLAR, J.L., CLUTTERBRUCK, R. & JINKS, S. (1983). Studies on the toxicity of cyclophosphamide in combination with mesna *in vitro* and *in vivo*. *Cancer Treat. Rev.*, **10**, suppl. A, 63.

MYERS, C.E. (1982). Anthracyclines. In *Pharmacologic Principles of Cancer Treatment*, Chabner, B. (ed) p. 416. Saunders: Philadelphia.

OZOLS, R.F. & COWAN, K. (1986). New aspects of clinical drug resistance: the role of gene amplification and the reversal of resistance in drug refractory cancer. In *Important Advances in Oncology*, DeVita, V.T., Hellman, S. & Rosenberg (eds) p. 129. J.B. Lippincott: Philadelphia.

PANNACCIULLI, I., MASSA, G., BOGLIOLO, G., GHIO, R. & SOBRERO, A. (1982). Effects of high dose methotrexate and leucovorin on murine hemopoietic stem cells. *Cancer Res.*, **42**, 530.

SUNDERMAN, F.W. (1971). The treatment of acute nickel-carbonyl poisoning with sodium diethyldithiocarbamate. *Ann. Clin. Res.*, **3**, 182.

TATKEN, T.L. & LEWIS, R.J. Jr. (1981/2). *Registry of Toxic Effects of Chemical Substances*, p. 823. United States Department of Health and Human Services, National Institute for Occupational Safety and Health: Cincinnati.

TILL, J.E. & McCULLOCH, E.A. (1961). A direct measurement of the radiosensitivity of normal mouse bone marrow cells. *Radiat. Res.*, **14**, 213.

TOMASHEFSKY, P., ASTOR, M. & deVERE WHITE, R. (1985). Relationship between thiol depletion and chemosensitization in a transplantable murine bladder tumor. *J. Natl Cancer Inst.*, **74**, 1233.

WASSERMAN, T.H., PHILIPS, T.L., ROSS, G. & KANE, L.J. (1981). Differential protection against cytotoxic chemotherapeutic effects on bone marrow CFU, by WR-2721. *Cancer Clin. Trials*, **4**, 3.

YODA, Y., NAKAZAWA, M., ABE, T. & KAWAKAMI, Z. (1986). Prevention of doxorubicin myocardial toxicity in mice reduced by glutathione. *Cancer Res.*, **46**, 2551.

YUHAS, J.M. & CULO, F. (1980). Selective inhibition of the nephrotoxicity of *cis*-dichlorodiammineplatinum (II) by WR-2721 without altering its antitumor properties. *Cancer Treat. Rep.*, **64**, 57.

YUHAS, J.M., SPELLMAN, J.M., JORDAN, S.W., PARDINI, M.C., AFZAL, S.M.J. & CULO, R. (1980). Treatment of tumors with the combination of WR-2721 and *cis*-dichlorodiammineplatinum (II) or cyclophosphamide. *Br. J. Cancer*, **42**, 574.